# Medical Clinic of Atlanta Medical College

**Published:** 1871

**Authors:** 


### *Medical Clinic of Atlanta Medical College—Service of Dr. J. G. Westmoreland.*

---

Case 1.—White girl æt 15.—Diagnosis. · *Chorea.*—She is not very perfectly developed for one of her age, and has never menstruated. Her mode of living is not most favorable to vigorous health.

This girl, as you remember, has been under treatment for a week, in the Dispensary, with improvement, as you perceive from her appearance to-day. She now speaks more distinctly, and has more control over movements of the extremities.

The first prescription was made with the view of giving tone and vigor to the spinal nervous centre, and consisted of five grains of quinine every six hours, until twenty grains had been taken, and the shower-bath every morning at sunrise or before. A decided improvement was visible in forty-eight hours, notwithstanding the error committed in using the shower. The water was used in too large quantity, and its application continued too long. The patient became thoroughly chilled, did not react readily, and was, therefore, depressed by the operation, rather than invigorated.

In the use of water, as a remedial agent, its temperature and manner of application are all-important for the accomplishment of any particular object. Hence, when invigoration is sought, it must be applied in a particular manner, else the opposite effect may be produced, as in this case.

In order to serve, as a nervous tonic, the water must be much cooler than the body, and applied suddenly, in quantity only sufficient to sprinkle the whole body at the same time, while in the erect position. The patient is then wiped and dressed at once. A very simple apparatus is sufficient for the purpose. In the absence of a regular arrangement for the shower-bath, a tin pan or box, the bottom being pierced with holes, can readily be obtained.

By continuing the shower some minutes, the temperature of the body is permanently reduced, and general depression of the vital energies is the result, but when restricted in the manner just described, it serves as a powerful invigorator, particularly to the nervous system.

Very impressible subjects require that the temperature of ordinary well or spring water be raised somewhat, for the first two or three applications, or until the patient has become accustomed to its use.

On the third day, in the treatment of this case, we called your attention to the reputation of bromine in the treatment of nervous disturbance of this character, and prescribed bromide of potassium in the dose of twenty grains, three times a day. This, without any other treatment, was continued two or three days, and as you observed, without any apparent benefit.

Two days ago, quinine, in the dose of five grains three times a day, was again prescribed, with properly conducted shower-bath and cups to the spine. The improvement, under this course of treatment, would seem to justify its continuance. The quinine will be suspended, however, till her return to the Dispensary, in a day or two, to be resumed, if necessary, then. The shower-bath will be continued regularly every morning.

Case 2.—A colored girl, æt 10 years. *Diagnosis*—Purpura hœmorrhagica.

This girl was brought to the Dispensary three days since. At that time blood was constantly oozing from the gums and tongue. The purplish spots on the skin, which have not yet entirely disappeared, were then very distinct.

Different theories have been given in explanation of this morbid phenomenon. That which attributes this hemorrhagic disposition to a modification of the blood itself, is perhaps most generally received.

It is, however, a well known fact, that a condition of the capillaries favorable to the effusion of blood, is the result of insufficient nervous influence, and it is believed that hemorrhage from this cause sometimes occurs.

Depending entirely, in the treatment of this case, upon remedies calculated to effect the circulating fluid directly, the muriated tincture of iron was given in the dose of ten drops every four hours, and the local application of solution persulphate of iron to the gums and tongue, as a temporary styptic. This treatment was continued for about two days, without any change in the symptoms, except cessation of hemorrhage from the mouth measurably, and its establishment in the bowels and kidneys, as seen in the evacuations of the bowels and in the urine.

On yesterday morning the use of iron was suspended, and carbolic acid, in the dose of a grain and a quarter in two or three ounces of water, was given every four hours. The improvement has been prompt and decided. No evidences of hemorrhage now exist, and convalescence will probably be rapid and the relief permanent.

Case 3.—A colored woman, æt 35 years. *Diagonis—Acute Rheumatism.*

In this affliction, various and opposite plans of treatment have been instituted, each having its advantages, and successful cases reported.

The alkaline, the acid, the anti-phlogistic and the neurotic plans are all sanctioned by popul ar authors and experienced practitioners. While one relies exclusively on vegetable acids, in the form of lemon juice, etc.; another claims equal success in the use of antacids, such as soda, lithia and potassa. This leads us to infer that entirely different, and even opposite primary pathological conditions exisit in the production of rheumatic inflammation, or that the disease subsides independently of treatment. A peculiar migratory feature of the affection leads us to believe that the latter may be the true explanation. It is found that in a few hours there is complete transfer of inflammatory symptoms from one portion of the body to another ; leaving the original location comparatively free from disease. This transfer is not always to a different portion of the same limb, but frequently from the knee or ankle to the wrist or elbow, or even the opposite side of the body. When such sudden changes occur it is not entirely imaginary to conclude that in leaving a part in this manner, from natural cause, the whole body may sometimes remain free from the disease, independent of the action of remedies, and that a reported result of treatment, may occasionally be only a coincidence.

It is difficult to believe that an excess of acid in the blood, and a dimunition below the healthy standard, will lead to the same result in the production of rheumatism. A very common error is committed in concluding that relief from disease is always the result of treatment.

Carefully conducted experiments, in several cases, with accurately recorded statistics, are necessary to the establishment of practical truths, in therapeutics.

Rheumatic inflammation differs essentially from that arising in traumatic or other causes of common inflammation. The migratory peculiarity before alluded to, and the freedom from tendency to result in suppuration, are characteristic features of rheumatism, which impress us with the idea that the local manifestation is not the result of mechanical or chemical lesion in the part, by external agencies or those transmitted through the blood.

This course of reasoning brings us to the conclusion that in derangement of the nervous system, an explanation of these symptoms may probably be found. We are strengthened in this opinion by the satisfactory termination of numerous cases while under treatment, by neurotic remedies. As in other important diseases, the true pathology of rheumatism has probably been determined by the experimental or accidental use of remedies whose physiological action is definitely known. Quinine, having been established as a tonic invigorator of the spinal nervous centre, and its promptness in the relief of rheumatism being confirmed by the experience of many practitioners, the legitimate inference is that rheumatism depends upon a depressed condition of the spinal nervous energies.

The distinction between rheumatism and its results, must be kept in view. From long continuance of acute articular rheumatism deposits of fibrin, which become semi-organized, and which interfere, for an indefinite time, with mobility of the joints, require iodine or other catalytics of fibrinous deposit. To the feet and ankles alone, the disease, in this case, seems to be confined. This patient will take five grains quinine every six hours till twenty grains are taken, with blistering to the spine.

